# Intraoperative Abdominal Penetration of the Lag Screw: A Rare Complication During Proximal Femoral Nail Anti‐Rotation Surgery

**DOI:** 10.1002/ccr3.72077

**Published:** 2026-02-14

**Authors:** Mohammad Javad Dehghani Firoozabadi, Ramin Bozorgmehr, Fatemeh Bastan, Maryam Rashidian

**Affiliations:** ^1^ Department of Orthopedic Surgery, Shahid Madani Hospital Alborz University of Medical Sciences Karaj Iran; ^2^ Department of General Surgery, Shahid Madani Hospital Alborz University of Medical Sciences Karaj Iran; ^3^ Student Research Committee, School of Medicine Alborz University of Medical Sciences Karaj Iran; ^4^ Alborz Office of USERN, Universal Scientific Education and Research Network (USERN) Alborz University of Medical Sciences Karaj Iran

**Keywords:** geriatric orthopedics, iatrogenic injury, intraoperative complications, PFNA, proximal femoral nailing, screw migration

## Abstract

Although the proximal femoral nail anti‐rotation (PFNA) is accompanied by several benefits, using the nail is technically challenging and may pose some errors, leading to osteosynthesis failure. Here, we report a critical presentation of an uncommon side effect of PFNA surgery. Intraoperative abdomen penetration via lag screw is a unique complication of PFNA surgery of which surgeons should be aware.

## 1 Introduction

The intertrochanteric femoral fracture in elderly patients is common with increased marked incidence in recent years as societies grow continuously older. Several clinical and biomechanical studies have evaluated different implants such as the dynamic hip screw (DHS), the Gamma nail (GN), and the proximal femoral nail (PFN) [1]. The proximal femoral nail anti‐rotation (PFNA) system is a new device introduced by the AO/ASIF in 2003. The PFNA implant includes a helical blade for improved antirotation stability [2].

The advantages of osteosynthesis using the PFNA include the option of either static or dynamic distal locking, an unreamed insertion approach, and good rotational stability of the head–neck fragment. Even though PFNA is technically challenging, it produces impressive results when used correctly and with the proper technique. The most vital elements for a successful outcome are a non‐varus reduction, correct nail insertion, and precise lag screw placement. A stable biomechanical reduction by closed, percutaneous, or open methods is essential for effectively managing unstable intertrochanteric fractures [3, 4, 5, 6].

However, the use of nails is technically difficult and carries a high risk of surgical error and complications. These can include: a damaged implant and non‐union 1 year after surgery; helical blade movement that ultimately led to pelvic perforation; helical blade penetration through the femoral head and ultimately result in osteosynthesis failure; and the hip screw's mechanical failure and loss of fracture fixation [7, 8, 9, 10].

Despite adherence to surgical principles (anatomic reduction, precise nail insertion, and optimal lag screw placement), critical failures may occur. Key risk factors for screw migration include osteoporotic bone, fluoroscopic misinterpretation, over‐reaming or eccentric screw trajectory, excessive screw length, and anatomic variants [11].

We present the first case of intra‐abdominal PFNA lag screw penetration during surgery, occurring despite real‐time fluoroscopy. This highlights the limitations of intraoperative imaging and the need for tactile feedback and multiplanar verification. This report has been reported in line with the SCARE criteria [12].

## 2 Case History/Examination

An 87‐year‐old woman was referred to emergency with chief complaints of hip trauma as a result of falling four stairs in the dark. Her past medical history was only significant for essential hypertension and coronary angiography. Her drug history was positive for daily atenolol and atorvastatin consumption. Her habitual, allergy, and past surgical history were negative. On examination, vital signs were detected to be within the normal range. Her right leg had no laceration. It was tender to touch, and she was unable to raise it. Distal pulses were palpable and in the normal range. Other limbs were normal on examination and not damaged.

## 3 Methods (Differential Diagnosis, Investigations and Treatment)

**FIGURE 1:**
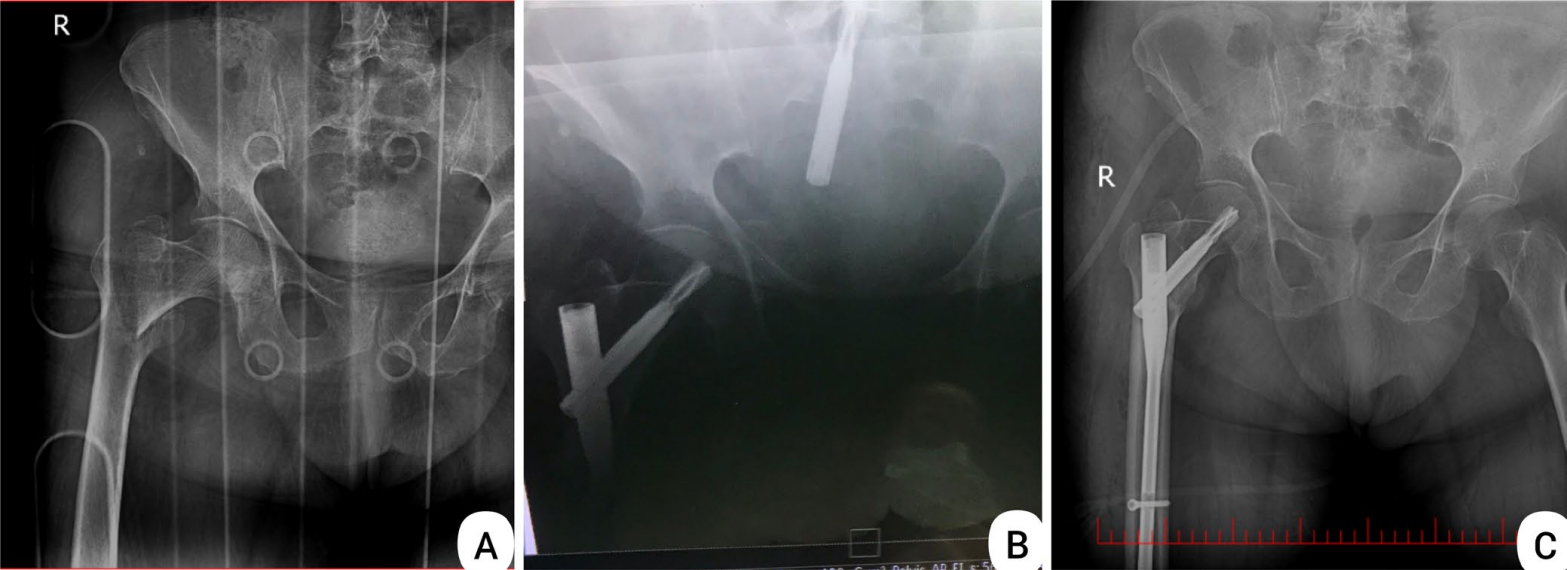
(A) Pre‐operative X‐ray of a right femur intertrochanteric fracture. (B) Intra‐operative X‐ray indicating the lag screw migrated to abdomen through acetabulum. (C) Post‐operative X‐rays of the fracture treated with proximal femoral nail.

**FIGURE 2:**
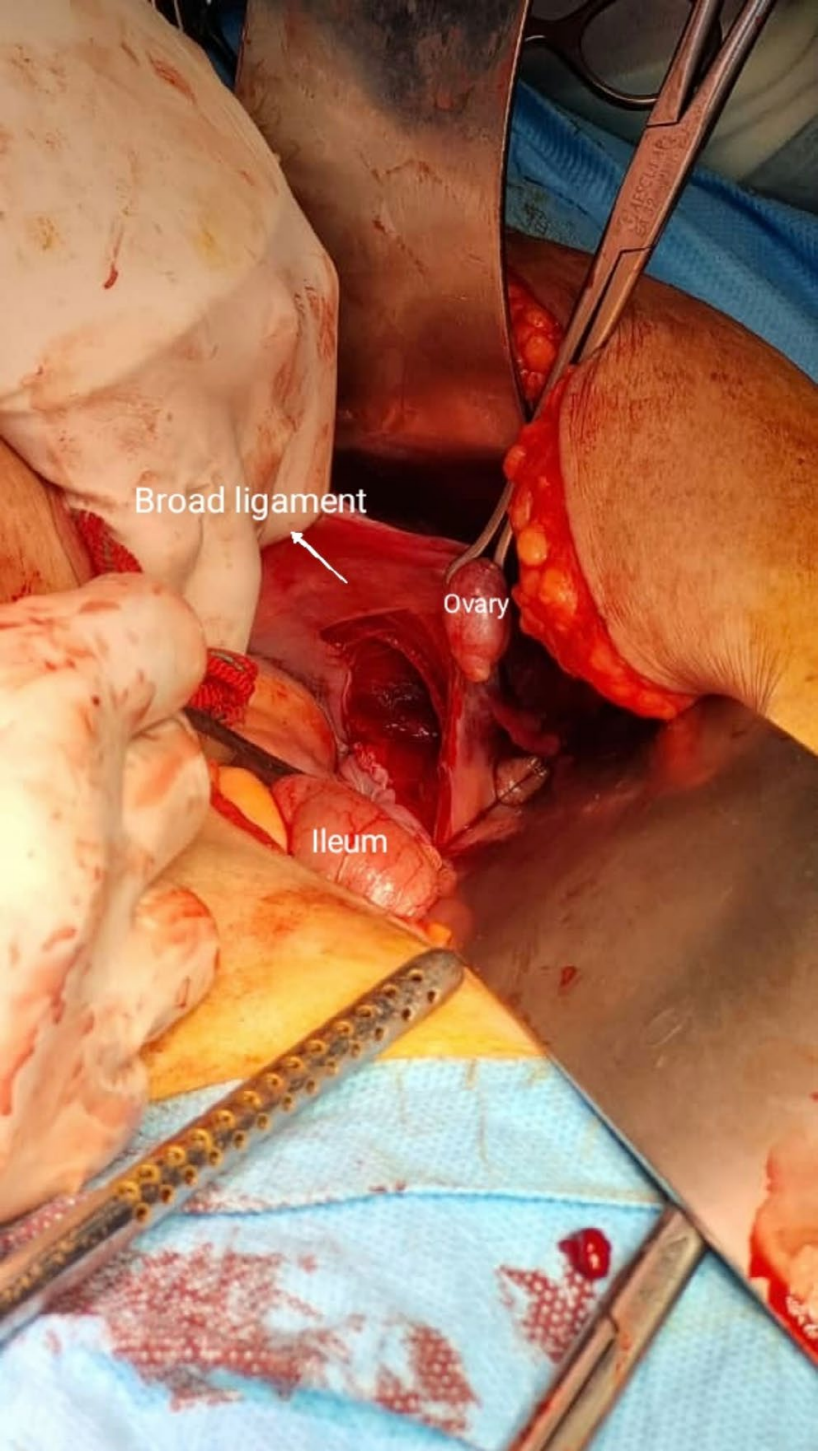
Exploratory laparotomy and removal of the nail. Despite penetration of the broad ligament, intra‐abdominal organs, including ileum, ovary, and ureter were not damaged.

Radiography revealed a right femur intertrochanteric fracture hip (AO/OTA classification 31‐A1) (Figure 1A). The patient was hospitalized. Intravenous heparin and acetaminophen as well as oral pantoprazole were initiated for her. Given the patient's preexisting cardiac disease and preoperative hemoglobin level of 9.1 g/dL, we administered 2 units of packed red blood cells based on our institutional protocol for high‐risk surgical patients. The surgery was performed under general anesthesia in supine position on a fracture table with manual traction. Antibiotic prophylaxis (cefazolin 1 g IV) was administered 30 min preoperatively. The procedure was conducted by a third‐year orthopedic resident under direct supervision of a senior trauma surgeon. During surgery, the femur was reset. The PFNA implant (manufactured by Mobtakran Co., Tehran, Iran) with a 240 mm nail length and 100 mm lag screw was placed. PFNA with a 240*12 mm short nail and 100 mm lag screw was placed with standard instructions. While fixing lag screw in the femur intraoperatively, we encountered unexpected screw migration into the abdominal cavity despite fluoroscopic guidance. It entered the abdominal cavity passing through the right acetabulum, iliopsoas muscle, and broad ligament (Figure 1B). Immediately, the general surgeon of the operating room performed exploratory laparotomy and removal of the lag screw. The ileum, ovary, ureter, and other intra‐abdominal organs hadn't been damaged (Figure 2). A new lag screw was placed successfully (Figure 1C). After recovery, she was transferred to the intensive care unit for 4 days and then moved to the orthopedic ward.

## 4 Conclusions and Results (Outcome and Follow‐Up)

The patient's postoperative trajectory was dominated by the intraoperative recognition of abdominal screw migration, a rare but critical complication. Immediate exploratory laparotomy confirmed the lag screw had penetrated the acetabulum and iliopsoas fascia without visceral injury. The surgical team successfully revised the fixation under augmented fluoroscopic guidance.

Subsequent recovery was complicated by thromboembolic events, including deep vein thrombosis (DVT) diagnosed on postoperative day 7 and pulmonary embolism confirmed by CT angiography. Anticoagulation management was further complicated by heparin‐induced thrombocytopenia (platelets 60,000/mm^3^), necessitating transition to direct oral anticoagulation. Despite stabilization of these conditions, the patient ultimately succumbed to pulmonary embolism on postoperative day 26 (Table 1).

**TABLE 1:** Timeline of clinical events.

Day	Event	Intervention
Day 0	Fall → Intertrochanteric fracture (31‐A1)	Hospitalization, IV heparin
Day 5	Surgery → Intraoperative screw migration to abdomen	Exploratory laparotomy → screw removal → successful revision
Day 5	ICU admission	Hemodynamic monitoring
Day 16	DVT diagnosis	Enoxaparin 60 mg SC BD
Day 17	Pulmonary embolism	
Day 21	HIT (Platelets: 60,000/mm^3^)	Switch to Apixaban Enoxaparin with prophylactic dose
Day 26		Discharge
Day 31		Resuscitation attempted

This case highlights three predictable risks in geriatric PFNA patients: (1) Thromboembolism: Due to immobilization and fracture‐associated hypercoagulability. (2) HIT: Secondary to heparin exposure. (3) Surgical complications: Osteoporosis and aberrant anatomy increased screw migration risk. Proactive measures (e.g., DOAC prophylaxis, preoperative CT pelvimetry) might mitigate these risks.

## 5 Discussion

Proximal femoral fractures and related operations are becoming more common, which leads to an increase in sequelae such as nonunion, osteonecrosis, loss of fixation, and peri‐implant femoral fractures [13, 14]. Although uncommon, proximal femoral nail failure can have severe consequences [15]. This case indicates the first report that the lag screw migrated to the abdominal region during PFNA surgery.

There are additional reports demonstrating the post‐operative nail migration. A fifty‐year‐old woman was described in one study as having a left pertrochanteric fracture, which was treated with proximal femoral nailing, and she was sent home. Nine months later, the woman returned to the emergency room complaining of pain and being unable to bear her weight. Imaging showed that the hip screw had mechanically failed and that the fracture fixation had been lost [10]. In another study, a 72‐year‐old man who had fallen and was experiencing excruciating hip pain visited Busan Veterans Hospital. An unstable trochanteric fracture of the right femur was revealed by plain radiography. He did not have any trauma, and two months following the PFN surgery, he presented with moderate to severe right hip discomfort. Radiographs revealed that the lag screw had punctured the pelvis through the acetabulum and femoral head [16]. Our study illustrated an 87‐year‐old woman with a right intertrochanteric femur fracture who underwent PFNA surgery, which resulted in the device entering her abdomen. Other similar cases to ours have reported post‐operative device displacement as a problem. However, in our instance, this issue arose during the procedure and no other report have been published such complication in the literature (Table 2).

**TABLE 2:** Cephalic screw migration cases.

Author	Year	Procedure	Treatment	Complications
Dimitrios Papanikolopoulos et al. [10]	2022	A 50‐year‐old woman was treated with proximal femoral nailing after she suffered a left pertrochanteric fracture. She returned nine months later, complaining of agony and being unable to support her own weight. Imaging demonstrated the lack of fracture fixation and the hip screw's mechanical failure.	A left hip hemiarthroplasty and the removal of the damaged hardware were part of the revision procedure	The hip screw's mechanical failure and the loss of fracture fixation
Prasoon Kumar et al. [8]	2024	A year after surgery, a 45‐year‐old man who had a subtrochanteric femur fracture that had been first treated with a short PFN was found to have a fractured implant and non‐union	The proximal nail portion was extracted using a typical technique during revision surgery. A beaded guidewire was used to retrogradely extract the distal portion through a lateral cortical window, avoiding the knee joint, while the central fragment was accessible and removed at the fracture site. Following the insertion of a long Proximal Femur Nail Antirotation (PFNA), bone grafting was carried out	A damaged implant and non‐union 1 year after surgery
Xiao‐Kun Chen et al. [7]	2022	An 84‐year‐old woman suffered an intertrochanteric fracture after falling at home had PFNA used as the intramedullary fixation device during surgery. The helical blade's medial migration, which ultimately resulted in pelvic perforation, was discovered during postoperative inspection.	The savage treatment we used was a cemented total hip arthroplasty. The patient experienced no pain or loosening of the left hip prosthesis at the most recent follow‐up, which was 12 months following total hip replacement	Helical blade movement that ultimately resulted in pelvic perforation
Mayur Nayak et al. [9]	2019	After a fall radiograph revealed an unstable left intertrochanteric fracture of the left hip, a 65‐year‐old man complained of excruciating left hip discomfort. PFNA‐II was used for internal fixation and a closed reduction. But 3 months after surgery, the patient complained of left hip pain when walking. The helical blade was perforated through the femoral head without any reduction loss, according to a follow‐up radiograph	After planning and executing a second surgery to remove the nail, it was discovered that the fracture had connected during the procedure	The helical blade's penetration through the femoral head

Even still, the exact reason for this event is still a mystery. This unusual complication may have been linked to some potential causes, including iatrogenic damage to the femoral head during reaming, improperly placed screws on the femoral head, early loading, exposing the implant to excessive torsional forces, direct trauma that results in a defect in the screw‐nail slot interface, misplacing the set screw in the proximal nail extremity, and selecting a cephalic screw that is either too short or too long [17, 18].

## 6 Conclusion

This case highlights a unique intraoperative complication of PFNA surgery—abdominal penetration by the lag screw and underscores the importance of meticulous surgical technique and immediate multidisciplinary intervention.

## Author Contributions

**Mohammad Javad Dehghani Firoozabadi:** data curation, investigation. **Ramin Bozorgmehr:** conceptualization, supervision, writing – review and editing. **Fatemeh Bastan:** visualization, writing – original draft. **Maryam Rashidian:** software, writing – original draft, writing – review and editing.

## Funding

The authors have nothing to report.

## Ethics Statement

All ethical and moral issues have been considered in this study.

## Consent

Written informed consent was obtained from the patient's legal next‐of‐kin after her passing, as documented in our institutional ethics committee records.

## Conflicts of Interest

The authors declare no conflicts of interest.

## Data Availability

The data that support the findings of this study are available on request from the corresponding author.
